# Massive hemoptysis and complete unilateral lung collapse in pregnancy due to pulmonary tuberculosis with good maternal and fetal outcome: a case report

**DOI:** 10.1186/1756-0500-6-335

**Published:** 2013-08-22

**Authors:** Gwinyai Masukume, Elton Sengurayi, Phinot Moyo, Julio Feliu, Danboy Gandanhamo, Wedu Ndebele, Solwayo Ngwenya, Rudo Gwini

**Affiliations:** 1 Department of Obstetrics and Gynaecology, Mpilo Central Hospital, Bulawayo, Zimbabwe; 2 Faculty of Medicine, National University of Science and Technology, Bulawayo, Zimbabwe; 3 Diagnostic X-Ray Centre, 101 Lancet House, P.O. Box 2428, Bulawayo, Zimbabwe; 4 Department of Peadiatrics, Mpilo Central Hospital, Bulawayo, Zimbabwe; 5 Department of Medicine, Mpilo Central Hospital, Bulawayo, Zimbabwe

**Keywords:** Tuberculosis, Pregnancy, Hemoptysis, Lung collapse, Health care worker

## Abstract

**Background:**

We report an extremely rare case of massive hemoptysis and complete left-sided lung collapse in pregnancy due to pulmonary tuberculosis in a health care worker with good maternal and fetal outcome.

**Case presentation:**

A 33-year-old human immuno deficiency virus seronegative African health care worker in her fourth pregnancy with two previous second trimester miscarriages and an apparently healthy daughter from her third pregnancy presented coughing up copious amounts of blood at 18 weeks and two days of gestation. She had a cervical suture *in situ* for presumed cervical weakness.

Computed tomography of her chest showed complete collapse of the left lung; subsequent bronchoscopy was apparently normal. Her serum β-human chorionic gonadotropin, tests for autoimmune disease and echocardiography were all normal.

Her lung re-inflated spontaneously.

Sputum for acid alcohol fast bacilli was positive; our patient was commenced on anti-tuberculosis medication and pyridoxine.

At 41 weeks and three days of pregnancy our patient went into spontaneous labor and delivered a live born female baby weighing 2.6 kg with APGAR scores of nine and 10 at one and five minutes respectively. She and her baby are apparently doing well about 10 months after delivery.

**Conclusion:**

It is possible to have massive hemoptysis and complete unilateral lung collapse with spontaneous resolution in pregnancy due to pulmonary tuberculosis with good maternal and fetal outcome.

## Background

Hemoptysis can be life threatening and its causes are many [1,2]. Tuberculosis is an important cause of mortality in pregnancy and can have profound effects on the unborn child and new born [3].

Complete lung collapse with massive hemoptysis in pregnancy is extremely rare [4,5].

We report an extremely rare case of massive hemoptysis and complete unilateral lung collapse in pregnancy due to pulmonary tuberculosis in a health care worker with good maternal and fetal outcome.

## Case presentation

A 33-year-old African health care worker pregnant for the fourth time with two previous second trimester miscarriages and an alive daughter from her third pregnancy presented to our emergency department at 18 weeks and two days of gestation, dated from the first day of bleeding of her last normal menstrual period, coughing up copious amounts of blood.

She had apparently been previously well until at about midnight on that day when she felt like clearing her throat then she coughed up frank blood that filled about half a small pale, the bleeding then stopped. She was afterwards feeling completely fine besides some shortness of breath. At about 19.00 hrs on that day while watching television with her husband she once again coughed up blood estimated to be about 500 ml whereupon she presented to our emergency unit.

Besides this episode she had never coughed up blood before, she denied history of coughing, night sweats, she did not smoke cigarettes, her last serologic test for HIV (human immuno deficiency virus) done three weeks previously was negative and besides being a health care worker, she had no known tuberculosis contact. She was experiencing shortness of breath and palpitations. A systemic inquiry focusing on the cardiovascular, gastrointestinal and central nervous systems was unremarkable.

She had a history indicated McDonald cervical suture inserted at 14 weeks of pregnancy for cervical weakness.

All her four pregnancies apparently had the same paternity. Her first two pregnancies ended as mid-trimester miscarriages. For her third pregnancy, cervical cerclage was performed and she subsequently delivered vaginally a live born female baby weighing 3 kg at term. Her five- year-old daughter was apparently in good health and attending early school.

Notably, she had been trying to conceive unsuccessfully while having regular unprotected sexual intercourse for more than one year.

She had no known illnesses or allergies and was taking iron and folate tablets.

Her family and social history was notable in that her sister was apparently asthmatic and was on treatment for this condition.

On examination, she was anxious and besides tachycardia her vital signs were normal. Whilst in the emergency department, she coughed up about 800 ml of frank blood. Examination of her chest revealed dullness to percussion on the left lower side and on auscultation there was decreased air entry and vocal resonance on the left side.

On obstetric examination, the height of fundus was about 18 weeks in size and fetal movements were noted.

She was resuscitated with rapid infusion of intravenous crystalloid, investigations were done and her previous medical records were reviewed (see Table 1 for the patient’s laboratory data).

**Table 1:** Laboratory data

**Variable**	**Reference range**	**Admission (18 weeks and 2 days)**	**12 weeks and 2 days**
White cell count (× 10^9^/L)	6–14	13.48	3.31
Hemoglobin (g/dL)	11.5–15.5	10.2	12.6
Platelets (× 10^9^/L)	150–400	209	271
Mean corpuscular volume (fl)	80–95	78	80.7
Sodium (mmol/L)	135–145	138	
Potassium (mmol/L)	3.6–5.0	4.0	
Urea (mmol/L)	3.2–6.7	2.6	
Creatinine (micromol/L)	53–115	126	
β-human chorionic gonadotropin (mIU/mL)	1000 – 100000	2375	

Echocardiography revealed normal mitral and tricuspid valves, normal left ventricular function and dimensions. There was no evidence of pulmonary hypertension. The ejection fraction was 68% (normal range 50% to 75%).

Ultrasound scan showed a single viable intra uterine pregnancy with a cephalic presentation at an estimated gestational age of 17 weeks. A left-sided cystic mass suspected to be of ovarian origin was noted. There was no evidence of molar pregnancy. Her first first trimester ultrasound scan had shown a left-sided adnexal mass (4.2 cm by 5.2 cm in size), it had been decided at that time to manage this mass conservatively.

**Figure 1:**
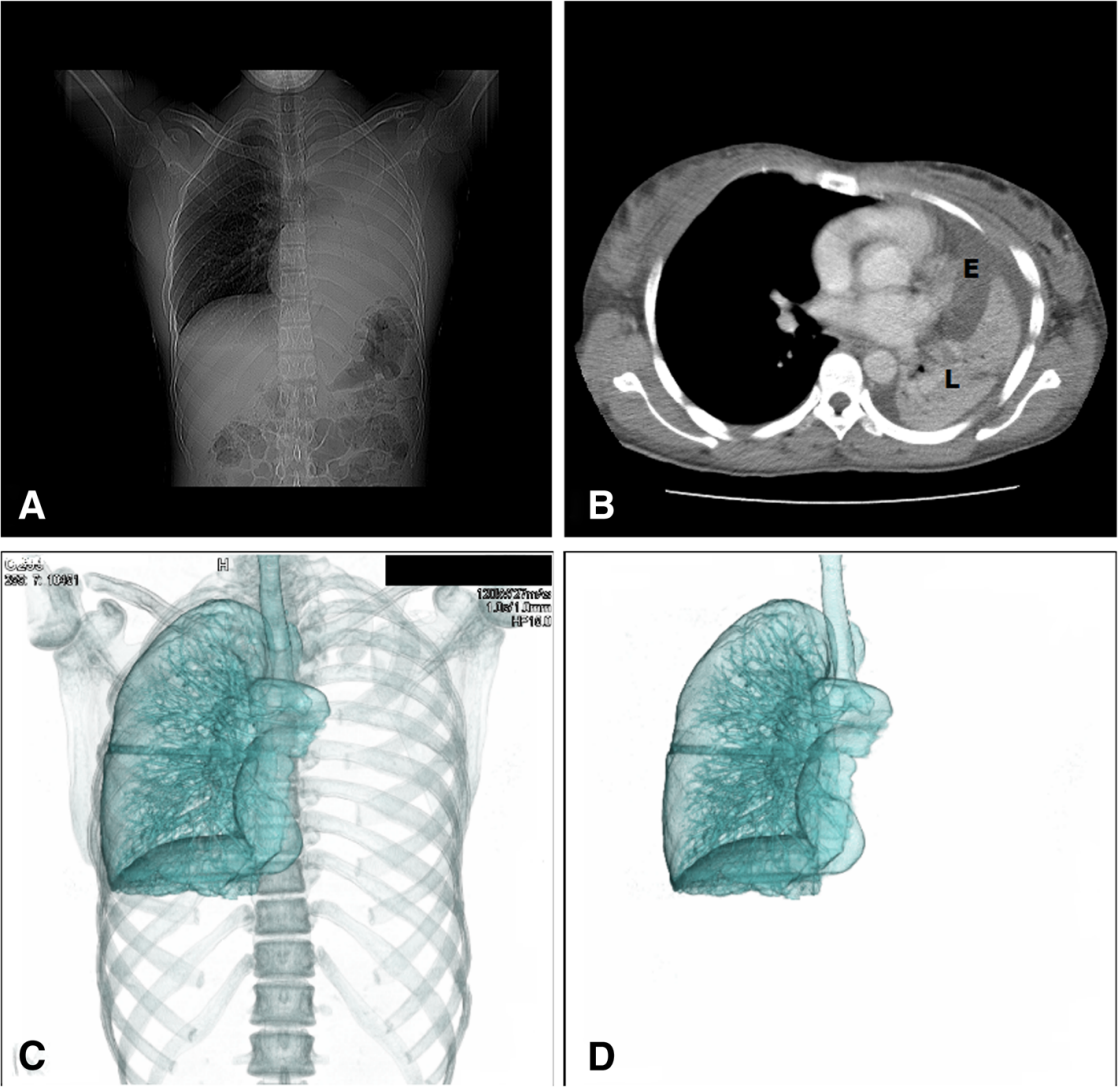
**Findings detected with computed tomography.** Panel **A**. Coronal CT of the chest showing left-sided lung collapse. Panel **B**. Axial CT of the chest revealing a pleural effusion (E) and the collapsed left lung (L). There is left mediastinal shift; the right lung is hyper-inflated. Panel **C**. Three dimensional (3D) reconstruction with volume rendering depicting complete collapse of the left lung with an obstructed left main bronchus. Panel **D**. Skeletal system removed from the 3D reconstruction.

Chest X-ray suggested left-sided lung collapse. Computed tomography (CT) of the chest with the administration of iodinated contrast material showed a left carinal mass with complete obstruction of the left proximal main bronchus 10 mm from the carina causing complete collapse consolidation of the left lung and a left pleural effusion (see Figure 1). Right middle lobe infiltrates were seen.

The radiologist was of the opinion that these findings were most likely due to tuberculosis or lymphoma. As metastases could not be excluded a bronchoscopy and biopsy was suggested. Rigid bronchoscopy was done; no abnormalities were seen.

Antiphospholipid antibodies, antibody to ds-DNA and antineutrophil cytoplasmic antibodies were all negative. Rapid plasma reagin was negative. Repeat serology for HIV three months after her first serologic test was negative, her partner was also seronegative.

Blood was also collected from the patient for coagulation studies; unfortunately the results were not available in time to inform her management.

She was initially admitted to the medical ward and then transferred to the intensive care unit (ICU). While in the ICU, she developed central chest pain and pyrexia. Her oxygen saturation ranged from 88 to 99% while she was receiving supplemental oxygen via nasal cannula. Her medications included ferrous sulphate, folate, intravenous crystal penicillin which was later switched to ceftriaxone, initially paracetamol then diclofenac. She also got steroids in the form of hydrocortisone at first then prednisone later. Intravenous crystalloids commenced from admission were continued.

**Figure 2:**
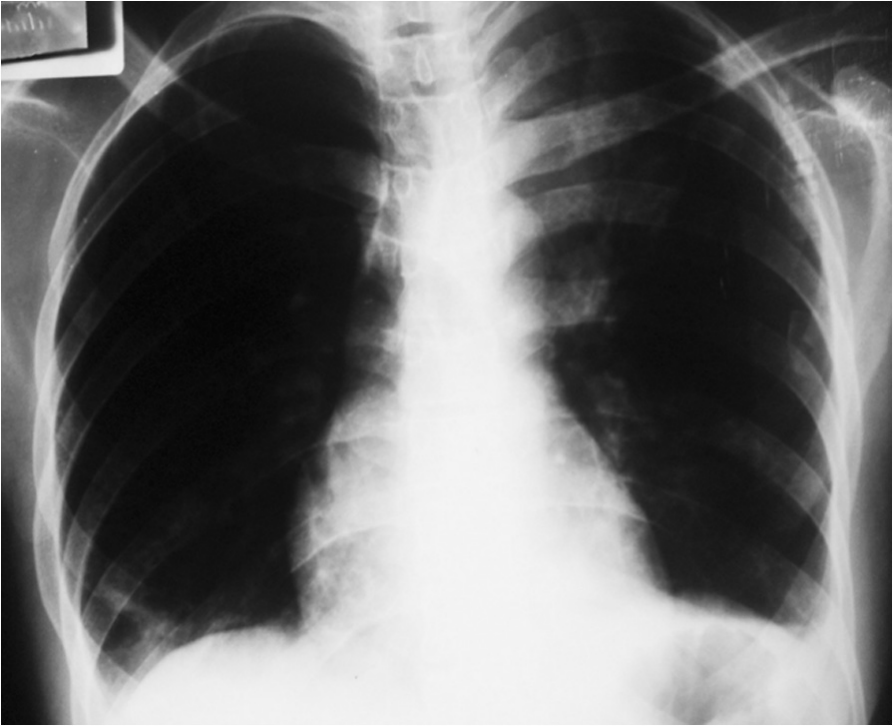
Chest X-ray showing left lung re-expansion.

Her pyrexia settled and her oxygen saturation improved progressively until she was saturating well on ambient air. Her left lung re-inflated spontaneously (see Figure 2); it was difficult to establish exactly when her lung re-inflated. Sixteen days after admission, she was discharged with a plan to continue antenatal care and to have chest physiotherapy while having further sputum specimen collection.

At 27 weeks and 1 day of pregnancy (about 9 weeks after admission) her direct sputum smear microscopy revealed acid alcohol fast bacilli and she was immediately commenced on anti-tuberculosis treatment (rifampicin, isoniazid, pyrazinamide and ethambutol) together with pyridoxine.

In summary, her antenatal care involved serial clinical examination and sonography. Her height of fundus was less than expected for her dates, for example, at 27 weeks gestational age, her symphysial fundal height was 21 cm which would correspond with a pregnancy of about 21 weeks.

Notably, her weight at 14 weeks of gestation (the time of cervical cerclage) was 55 kg; at 27 weeks, 55.7 kg; at 35 weeks 62.5 kg and at term 63.1 kg. She was 1.72 m tall.

**Figure 3:**
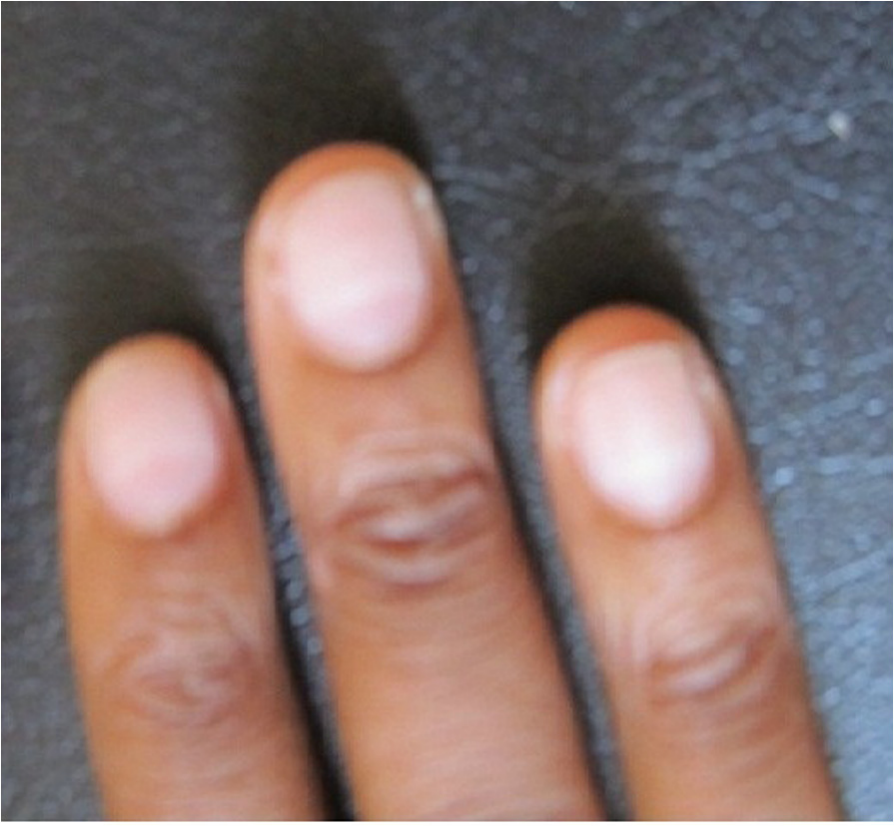
**White transverse nail lines at 27 weeks of gestation; nail abnormalities can provide important clues about systemic disease **[6]**.**

It was noticed that our patient at 27 weeks of pregnancy had white transverse nail lines (see Figure 3).

Her cervical suture was removed and at 41 weeks and three days of pregnancy she went into spontaneous labor, she delivered a live born female baby vaginally weighing 2.6 kg with APGAR scores of nine and 10 at one and five minutes respectively.

Our patient completed her six months of anti-tuberculosis treatment. Sputum collected during her anti-tuberculosis treatment for microscopy as well as sputum culture was all negative.

She and her baby are apparently doing well about 10 months after delivery. At the 10 month review, the infant’s growth and development were normal.

## Discussion

Hemoptysis may be defined as expectoration of blood that emanated in the lungs or bronchial tree [1]. Our patient had massive hemoptysis (greater than 200 ml in 24 hours).

The differential diagnosis of hemoptysis in pregnancy includes tuberculosis, bronchiectasis, pneumonia, aspergillosis, lung abscess, endobronchial lesions, tracheobronchitis, pulmonary embolism, bronchogenic carcinoma, carcinoid tumor, bleeding disorders, pulmonary vasculitis, arteriovenous malformations, amniotic fluid embolism, mitral valve stenosis and trauma [2]. A more comprehensive list of hemoptysis causes can be found in a review on the subject [1].

Our investigations were tailored to narrow these wide differential diagnoses. Another cause of hemoptysis that deserves specific mention is gestational trophoblastic disease [7]; this prompted us to ascertain the patient’s serum β-human chorionic gonadotropin levels, which were normal. As autoimmune phenomena can cause hemoptysis and miscarriages [8] our patient who had a history of previous miscarriages was also worked up for autoimmune conditions. The work-up was negative.

Complete blood count, serum electrolytes together with urea and creatinine, chest X-ray and CT, echocardiography, bronchoscopy and sputum for acid alcohol fast bacilli helped to pinpoint the cause of her massive hemoptysis.

We speculate that our patient was bleeding because of tubercular blood vessel erosion from bronchial or other systemic arteries supplying the lungs (high pressure system) rather than the pulmonary arteries and their branches (low pressure system). Due to the hypercoagulable state of both pregnancy and tuberculosis [4] we suspect that a blood clot formed (the left carinal mass seen on chest CT) and obstructed the left main bronchus resulting in complete left lung collapse. As far back as 1929 Wilson described a case of a patient with tuberculosis and lung collapse because of endobronchial obstruction caused by a blood clot [9].

There have been previous very rare case reports of pregnant women developing total lung collapse due to hemoptysis [4,5].

We also speculate that in our case, the blood clot disintegrated or dislodged spontaneously leading to recovery of the patient.

Imaging is particularly critical in the acute setting to prevent diagnostic delay that may cause maternal and fetal morbidity and mortality.

The typical conceptus dose from a posteroanterior chest X-ray is 0.002 milli Grays (mGy) and that from a standard dose level routine chest CT is 0.2 mGy. The risk of miscarriage, major malformation or malignancy is negligible in fetuses exposed to 50 mGy or less and the developing fetus is most susceptible to radiation effects between 8 to 15 weeks of gestational age [10]. In our case, the developing fetus at more than 18 weeks gestational age was exposed to far less than 50 mGy. Furthermore, no teratogenic effects have been reported with iodinated contrast materials [10].

The anti-tuberculosis drugs rifampicin, isoniazid, pyrazinamide and ethambutol have an excellent safety profile in pregnancy and are apparently not associated with human fetal malformations [11]. Our patient also received pyridoxine (Vitamin B6) as this is the recommended practice in pregnant patients also receiving isoniazid.

Some authorities recommend breastfeeding of infants if the mother is deemed not to be contagious and she has been receiving anti-tuberculosis medication for two or more weeks [3].

Diagnosis of tuberculosis in pregnancy can be challenging [3]; furthermore, up to a third of patients with hemoptysis may have no apparent underlying cause [1]. Small for dates, sub optimal weight gain and being a health care worker (higher risk of tuberculosis [12]) are features in our patient that could have helped make the diagnosis of tuberculosis sooner. However, establishing a definitive diagnosis of tuberculosis in pregnancy usually takes time [13].

Although we did not have histopathologic evidence our patient most likely had endobronchial tuberculosis (EBTB) which has a female preponderance and is more common in young adults [14]; our patient was a young adult female.

Sputum positivity in EBTB can range from 16% to 53% [15]; this fact may explain the initial sputum negativity in our patient. Bronchial biopsy which can be positive in 30% to 84% of patients [15] could have been done during bronchoscopy, however, biopsy could have been dangerous in a patient who may have had an underlying undiagnosed bleeding tendency.

Low birth weight and pre-term labor [11] are some of the complications of tuberculosis in pregnancy which we fortunately did not see in our case. Congenital tuberculosis is another complication - albeit rare - that we did not observe [16]. No special treatment was given to the new born as at the time of birth the mother had completed the intensive phase of anti-tuberculosis treatment and her sputum was negative for tuberculosis.

Among other medications, our patient received ferrous sulphate and corticosteroids. Attempting to maintain normal iron levels in tuberculosis patients may improve clinical outcomes [17]. The use of corticosteroids in EBTB may be beneficial [14] and perhaps for all forms of tuberculosis [18].

## Conclusion

It is possible to have massive hemoptysis and complete unilateral lung collapse with spontaneous resolution in pregnancy due to pulmonary tuberculosis with good maternal and fetal outcome.

## Consent

Written informed consent was obtained from the patient for publication of this case report and any accompanying images. A copy of the written consent is available for review by the Editor-in-Chief of this journal.

## Competing interests

The authors are all health care workers and may be at increased risk of developing tuberculosis.

## Authors’ contributions

RG was the patient’s primary physician. GM wrote the first draft of the article, inserted the cervical suture and was involved in the patient’s antenatal care. ES was involved in the patient’s antenatal care. PM anesthetized the patient. JF did the bronchscopy. DG was the radiologist. WN was the pediatrician. SN was the obstetrician and gynecologist overseeing all obstetric issues. ES, PM, JF, DG, WN, SN and RG revised the manuscript making important intellectual contributions. All authors read and approved the final version of the manuscript.
